# Longitudinal functional brain connectivity maturation in premature newborn infants: Modulatory influence of early music enrichment

**DOI:** 10.1162/imag_a_00373

**Published:** 2024-11-18

**Authors:** Annemijn Van Der Veek, Serafeim Loukas, Lara Lordier, Joana Sa de Almeida, Manuela Filippa, François Lazeyras, Dimitri Van De Ville, Petra S. Hüppi

**Affiliations:** Division of Development and Growth, Department of Woman, Child and Adolescent, University of Geneva, Geneva, Switzerland; Department of Psychology and Educational Sciences, University of Geneva, Geneva, Switzerland; Department of Radiology and Medical Informatics, University of Geneva, Geneva, Switzerland; CIBM, Center of Biomedical Imaging, Geneva, Switzerland; Neuro-X Institute, Ecole Polytechnique Fédérale de Lausanne, Geneva, Switzerland

**Keywords:** prematurity, resting-state fMRI, connectome analyses, longitudinal maturation, music intervention

## Abstract

Premature birth affects brain maturation, illustrated by altered brain functional connectivity at term equivalent age (TEA) and alters neurobehavioral outcome. To correct early developmental differences and improve neurological outcome, music during the neonatal intensive care unit (NICU) stay has been proposed as an auditory enrichment with modulatory effects on functional and structural brain development, but longitudinal effects of such interventions have not been studied so far. We longitudinally investigated resting-state functional connectivity (RS-FC) maturation in preterm infants (n = 43). Data-driven Independent Component Analyses (ICA) were performed on scans obtained at 33- and 40-week gestational age (GA), determining the presence of distinct resting-state networks (RSNs). Connectome analysis “accordance measure” quantitively examined the RS-FC both at 33- and 40-week GA. Further comparing the internetwork RS-FC at 33- and 40-week GA provided a circuitry of interest (COI) for significant maturational changes in which the effects on the RS-FC of a music intervention were tested. The connectome analyses resulted in a COI of RS-FC connections significantly maturing from 33 to 40 weeks GA, namely between the thalamic/brainstem and prefrontal–limbic, salience, sensorimotor, auditory, and prefrontal cortical networks; between the prefrontal–limbic and cerebellar, visual and left hemispheric precuneus networks; between the salience and visual, and cerebellar networks; and between the sensorimotor and auditory, and posterior cingulate/precuneus networks. The infants exposed to music exhibited significantly increased maturation in RS-FC between the thalamic/brainstem and salience networks, compared with controls. This study exemplifies that preterm infant RS-FC maturation is modulated through NICU music exposure, highlighting the importance of environmental enrichment for neurodevelopment in premature newborns.

## 1 Introduction

The emergence of brain resting-state networks (RSNs) in the fetus and the newborn occurs during the period of rapid brain development over the third trimester of gestation; intra- and extrauterine studies have unveiled that at the end of the second trimester up until 33 weeks gestational age (GA), spontaneous neural activity organizes in regions for primary sensory functioning, such as in visual, auditory, somatosensory, and motor networks (De Asis-Cruz et al., 2020;Doria et al., 2010;He & Parikh, 2016;Jakab et al., 2014;Rajasilta et al., 2020;Schöpf et al., 2012;Smyser et al., 2010;Turk et al., 2019). Fragmentary elements for higher order cognitive functions also develop: the posterior cingulate cortex (PCC) and precuneus form a primitive default mode network (DMN) (He & Parikh, 2016;Hu et al., 2022;Scheinost et al., 2024;Seshamani et al., 2016), which throughout life involves in cognitive capacities such as self-reflection, theory of mind, mind wandering, and executive control (Buckner et al., 2008;Grady et al., 2006;Raichle et al., 2001;Seeley et al., 2007;Sherman et al., 2014). Further, frontal and parietal cortical networks emerge for executive control, pertinent for sustained attention and working memory (Doria et al., 2010;He & Parikh, 2016;Hu et al., 2022;Scheinost et al., 2024;Schöpf et al., 2012;Smyser et al., 2010;Thomason et al., 2013,^2015^). The salience network comprises the insula and anterior cingulate cortex (ACC), with a functional role for relevance distinction from internal and external stimuli and identifying pertinent action (Canini et al., 2020;Li et al., 2019;Seeley et al., 2007). In- and ex-utero studies have detected that insular activity arises prior to 33 weeks GA (Canini et al., 2020;Ji et al., 2022;Scheinost et al., 2024), with ACC resting-state activity found correlated with the insula (De Asis-Cruz et al., 2020). In addition, resting-state activity also emerges in subcortical regions, involved in stress regulation and emotion processing (De Asis-Cruz et al., 2020;Wu et al., 2018;Xiao et al., 2018). The spontaneous neural activity required for the establishment of neural networks arises at a time when thalamocortical projections are developing, which indicates that the spatially distributed correlations that form RSNs may be linked to thalamocortical activity (Kostovic & Jovanov-Milosevic, 2006;Zhang et al., 2010). With increasing GA, global efficiency improves through RSN clustering (Cook et al., 2023). At the time of term age, a full higher order infrastructure for the DMN, the executive control network, and the salience network has been shown to be present (Cao et al., 2016;Doria et al., 2010;Hu et al., 2022;Lordier, Meskaldji, et al., 2019;Rajasilta et al., 2020;Scheinost et al., 2024;Smyser et al., 2010).

Interestingly fetuses that end up being born prematurely already display weaker resting-state connectivity prior to birth (Thomason et al., 2017) and the ex-utero early life experiences of the preterm infant further modify RSN development as shown by altered brain resting-state functional connectivity (RS-FC) at term equivalent age (TEA) when compared with full-term newborns (De Asis-Cruz et al., 2020;Doria et al., 2010;Eyre et al., 2021;Hu et al., 2022;Pino et al., 2023;Smyser et al., 2016,^2010^;Thomason et al., 2017;Toulmin et al., 2015;Zhang et al., 2019). More specific, RSN disruption due to prematurity is revealed by a globally reduced within-network RS-FC for areas involved in primary sensory functioning and association areas (Eyre et al., 2021). Furthermore, resting-state thalamic integration of cortical regions is negatively affected by prematurity for areas involving the insula, ACC, and prefrontal networks at TEA (Toulmin et al., 2015), which underlines the importance of this period for the integration of thalamocortical connectivity. In addition, the salience network is found to be impaired in its function as a central hub, impacting the connectivity among other circuits (Lordier, Meskaldji, et al., 2019). A reduction in lateralization difference of the resting-state structure is also assessed at TEA for DMN and executive control networks (Kwon et al., 2014). Further RS-FC alterations after premature birth have been shown to persist to childhood and adolescence (Damaraju et al., 2010;Wehrle et al., 2018). RS-FC contributes in understanding the basis of neurodevelopmental outcome of preterm infants with correlations of RS-FC strength and functional performance in both motor and cognitive domains (Jakab et al., 2020;Toulmin et al., 2021). Taken together, early neurodevelopment and plasticity is an important stage for laying the foundation for future mental functioning (Cho et al., 2022;Papini et al., 2016;Siffredi et al., 2022).

Music interventions have recently been proposed for modulating the development of preterm infants during their neonatal intensive care unit (NICU) stay with wide range effects on physical and behavioral measures (Bieleninik et al., 2021;Filippa et al., 2020) as well as direct effects on brain structural (Sa de Almeida et al., 2020,^2023^) and functional integrity. After NICU musical enrichment, premature infants at TEA have displayed RS-FC strength more similar to full-term newborns (Lordier, Meskaldji, et al., 2019;Loukas, Lordier, et al., 2022). In particular, significantly higher connectivity is found between the salience network as central hub and superior frontal, auditory, sensorimotor, thalamic, and precuneus networks (Lordier, Meskaldji, et al., 2019). Modulation of resting-state activity at TEA is shown after music listening as well, that is, within networks involved in familiarity processing and associative memory retrieval (Loukas, Lordier, et al., 2022). While these results are promising, there is lacking evidence of direct intraindividual effects of music interventions. A valuable addition for studying the effects of music as NICU environmental enrichment on resting-state maturation, therefore, is assessing the differences longitudinally from prior to the intervention to after the intervention.

## 2 Materials and Methods

### 2.1 Cohort

Seventy (n = 70) preterm infants with a mean birth gestational age of 29.06 weeks (SD = 2.2) were recruited at the University Hospital Geneva, Geneva, Switzerland, after the parents provided informed consent. The premature infants were followed longitudinally with two MRI scans: one at 33 weeks GA and one at TEA/40 weeks. The 70 preterm infants were randomly assigned to receiving a music or no music during their NICU stay. For the 70 recruited infants, 10 participants dropped out prior to the first MRI scan. Of the 60 remaining infants, the music intervention group consisted of 32 infants, whereas 28 were part of the control group. Five participants did not receive a scan at TEA/40 weeks GA after the music intervention, due to dropout or COVID-19 (seeFig. 1).

**Fig. 1.**
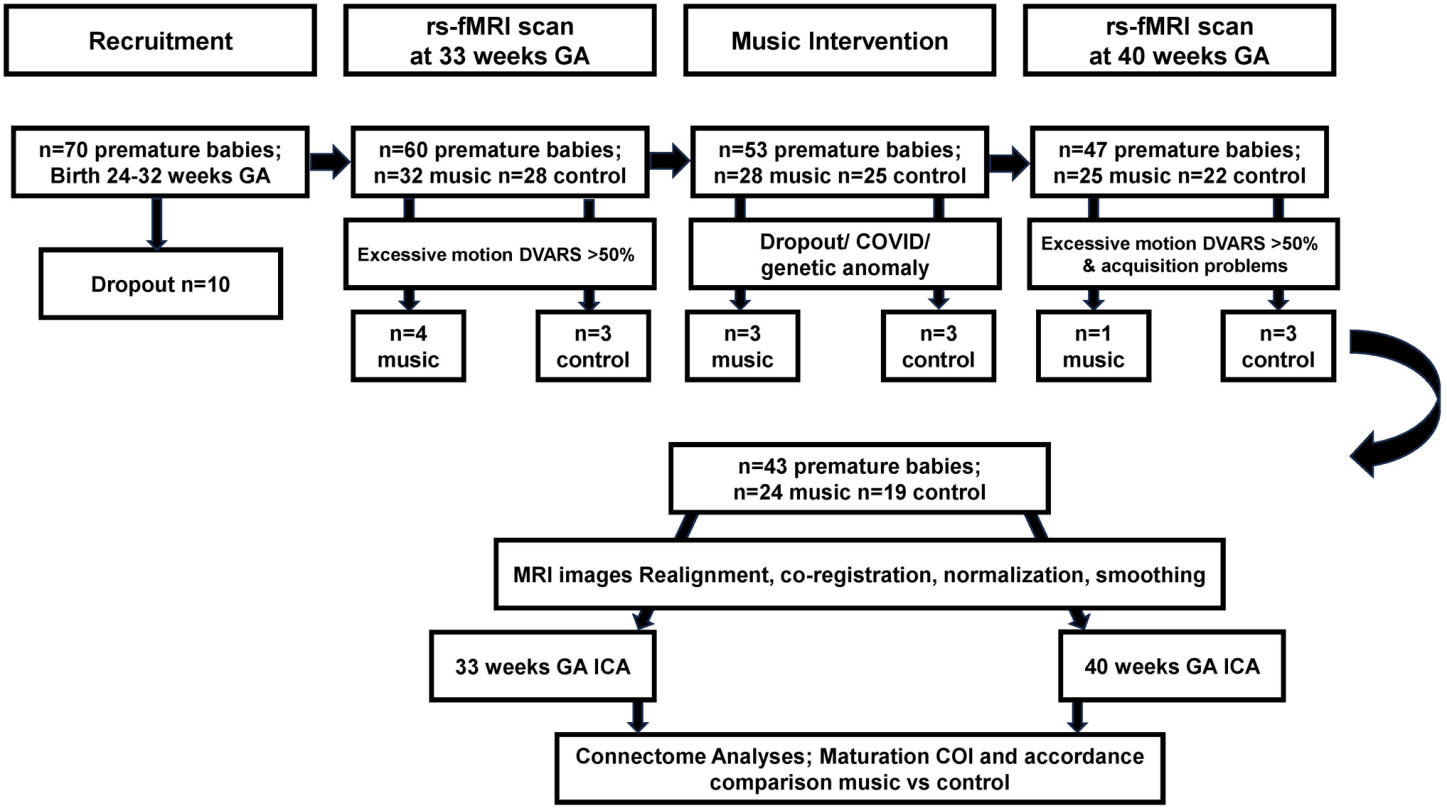
Flowchart of sample recruitment, data acquisition, preprocessing, and analyses within the premature infant music intervention study.

Inclusion criteria of the study was preterm birth prior to 32 weeks of gestation, no major brain lesions detected on MRI, such as high-grade intraventricular hemorrhage or leukomalacia. One baby was excluded from further analysis due to detected genetic anomaly. In addition, MRI frames with excessive motion were excluded (see 2.4 Preprocessing section for more details). Seven babies for this reason were excluded from the first scan session prior to the music intervention (4 in the preterm music group and 3 in the preterm control group), and 2 were excluded from the 40 weeks GA time point (1 in the preterm music group and 1 in the preterm control group). In addition, of the babies that had a scan prior to the intervention, 2 subjects were not usable in the control group due to acquisition problems. The full longitudinal analysis was, therefore, possible on 43 babies. In total, 24 babies were part of the music intervention group (10 females, mean (SD) GA at birth: 29.2 (1.9) weeks), with a scan prior to the music intervention at (mean GA (SD) at scan 1) 33.7 (0.5) weeks, and at TEA after the intervention at (mean GA (SD) scan 2) 40.1 (0.6) weeks. In total, 19 babies were part of the control group (12 females, mean GA (SD) GA at birth: 28.9 (2.5) weeks), with a scan prior to the music intervention at (mean GA (SD) at scan 1) 33.7 (0.4) weeks, and at TEA after the intervention at (mean GA (SD) scan 2) 40.1 (0.5) weeks.

No statistically significant differences (based on analyses of variance (ANOVA) test for numerical variables and chi-squared test for categorical variables) were observed for premature music and control group characteristics based on GA at birth, weight, height, and head circumference, gender, twin, sepsis (positive blood culture), bronchopulmonary dysplasia, mean number of headphones interventions, and GA at scan 1 and scan 2 (Population characteristics and statistical tests are described in theAppendix, and presented inAppendix Table A1).

### 2.2 Music intervention design

The music interventional trial was designed as a double-blinded randomized controlled trial. The specifically created musical composition was applied through wireless headphones through a protocol approved by the state ethical committee on human research. The intervention was based on three musical pieces composed by Andreas Vollenweider and consisted of instrumental melodies (with bells, harp, punji (charming snake flute)) each adapted to the three distinct behavioral states of the infant (falling asleep, waking up, being awake). The music composition and choice of instruments were based on behavioral responses, as observed by a trained nurse using a sensory-motor scale for preterm infants. All babies received the three musical pieces during their stay in neonatology, distributed according to the vigilance state of the moment. The music was played only before or after the infants received a care procedure (diaper changes, feeding, etcetera). The music was administrated using headphones, and the control group also received headphone administration, with sole difference being that they did not hear any music via the headphones. The headphones in the control group were modified with the aim not to muffle the surrounding sound. The differences in maturation observed between the music and control groups cannot, therefore, be explained by sound deprivation, but rather by the impact of music. The protocol is described in detail byLordier, Meskaldji, et al. (2019)who reported results from an earlier cohort. The intervention commenced when the infants were 33 weeks GA, and the duration lasted until hospital discharge.

The infants in the music group received the headphones on average 1.96 times per day (mean number of 55.8 (SD 24.9) times over mean days 28.4 (SD 10)), and the headphones were administered on average 2.24 times daily in the control group (mean number of 58.1 (SD 28.4) times over mean days 25.9 (SD 10.6)). All adults surrounding the care, meaning the parents, caregivers, and music intervention providers, were blinded to the group assignment.

### 2.3 MR image data acquisition

All infants were scanned after receiving breast or formula feeding, during natural sleep (no sedation used). At time point 1, preterm infants were scanned using an MR-compatible incubator built by Lammers Medical Technology (Lübeck, Germany) and monitored using Philips MR patient monitor Expression MR400 equipped with Philips quadtrode MRI neonatal electrocardiogram (ECG) electrodes. At time point 2, infants were scanned using a vacuum mattress for immobilization. All infants, at both time points, were monitored for heart rate and oxygen saturation during the entire scanning time. MR-compatible headphones were used (MR confon, Magdeburg, Germany) to protect infants from the scanner’s noise. MRI acquisition at both time points was performed on 3.0T Siemens MR scanners (Siemens, Erlangen, Germany) Siemens Magnetom, using a 16-channel neonatal head coil. T2-weighted structural images were acquired using the following parameters: A T2*-weighted structural image for anatomical reference (TR = 4990 ms, TE = 151 ms, flip angle = 150°, matrix = 256 x 164; voxel size = 0.78 x 0.78 x 1.2 mm^3^). During a 7-minute run of rs-fMRI data acquisition, the fMRI data were obtained by means of T2*-weighted gradient-echo echo planar imaging (EPI) images with the following parameters: 590 images, TR = 700 ms, TE = 30 ms, 36 slices, voxel size = 2.5 x 2.5 x 2.5 mm^3^, flip angle = 60°, multiband factor = 4.

### 2.4 Preprocessing

The data acquired was preprocessed using SPM12 (Wellcome Department of Imaging Neuroscience, University College London, United Kingdom). The preprocessing steps included realignment; adjusting for motion at the beginning and end of the scan, coregistration; alignment in Montreal Neurological Institute (MNI) space and overlay of fMRI data from a single subject and correlate with that subject’s own but separately acquired anatomic images, and normalization; in which all the scans prior to and after the music intervention are warped to a 40-week GA template. The template used to compare the 33 weeks with the 40 weeks GA scans was derived from 20 full-term and preterm at TEA infants, from which we selected the best quality 40 weeks GA reference image. Using a 40 weeks GA template provided sufficient precision also for the 33 weeks GA scans. Slice-timing correction was omitted due to a fast TR of 700 ms. Finally, the data were smoothed with a Gaussian filter of full width at half maximum (FWHM) of 6 mm. For motion correction, the volumes with a framewise displacement (FD) higher than 0.5 mm or with a rate higher than 3% of BOLD signal changes across the entire brain were removed. This measure mitigates the effect of the difference in shape and volume by how much the intensity of a brain image changes in comparison with the previous time point (as opposed to the global signal, which is the average value of a brain image at a time point), and is called the temporal derivative of time courses and variance in voxels (DVARS) (Power et al., 2014). Along with this, the previous and the two following images were eliminated. The remaining images were used for the analysis. Sufficient for inclusion is a minimum of 50% of volumes remaining. For seven preterm infants, these requirements were not met (four in the preterm music group, and three in the preterm control group). The average number of remaining images after the process of elimination for the two time points is presented inAppendix Table A1.

### 2.5 Group ICA to define resting-state networks

A group-level independent component analysis (group-ICA) was conducted to extract independent spatial networks, and these components were thresholded at z-score of 2. The results were obtained in a single group-level ICA using the GIFT toolbox in MATLAB. A mask composed of full-term and preterm newborn template segmentation was implemented to remove voxels in the cerebrospinal fluid, ventricles, eyes, and extracerebral areas (skull). The ICA was repeated 20 times using ICASSO for stability of the decomposition and determined 13 robust components when combining the 33- and 40-week GA scans. Among the 13 components from the combined scans before and after the intervention, 2 reflected areas related to motion and blood vessels, rather than resting-state activity. These were considered noise and excluded from further analyses. The 11 remaining components were used as regions of interest (ROIs) to build the functional connectomes. A time course for each subject and each network was extracted by using MarsBar from the SPM toolbox.

### 2.6 Resting-state functional connectivity estimation

The functional connectivity was estimated by means of a nonparametric estimator, namely “accordance” (Meskaldji et al., 2016a). Accordance quantitatively estimates the functional connectivity between RSNs. The term aims to describe the coupling between the time courses of two resting-state ICA networks. This coupling represents coactivation and codeactivation (meaning coherently active RSNs) among ROIs, where both are obtained by thresholding the normalized fMRI signal at positive thresholds (for more details seeMeskaldji, et al., 2015,2016a). To specify the thresholds, the 80% quantile of the normal distribution was used, this for its robustness and consistency in line with the conventional Pearson’s correlation coefficient. This methodology has been considered more robust than the Pearson’s or Spearman’s correlation coefficient (Meskaldji et al., 2016b). For each subject in the experimental and control group, an RS-FC based on the coactivation accordance measure was derived, which ultimately defined the averaged preterm group ICA functional connectome (ICA-FC). The accordance measure has been effectively used in predictive studies (Lin et al., 2018;Yoo et al., 2018), as well as for group comparisons among preterm and full-term infants at term (Lordier, Meskaldji, et al., 2019).

### 2.7 Connectome-based statistical analysis

#### 2.7.1 Circuitry of interest: The RS-FC maturation from 33 to 40 weeks GA

The circuitry of interest (COI) was defined by statistically testing the differences between the preterm infants’ 33 weeks GA ICA-FC and 40 weeks GA ICA-FC. The statistical test, a paired t-test, was applied at each individual connection. The p-values were corrected for multiplicity using Benjamini–Hochberg false discovery rate (FDR) correction at an α-level equal to 0.05, since this method is better suited for exploratory analyses. Of these connections, the ones passing the FDR correction were identified as the “maturating COI”.

### 2.8 Effect of music on maturation from 33 to 40 weeks GA

To test the hypothesis that NICU music exposure has resulted in higher functional connectivity strength, the “maturating COI” was used to compare alterations in the premature music and control group ICA-FC from 33 to 40 weeks GA. We computed the RS-FC difference between the music and the control group based on a technique previously used for connectome analysis (Loukas, Lordier, et al., 2022), which is based on the conversion of the p-values from the t-tests into z-scores (Z premature music (Z_PM_), Z premature control (Z_PC_)). By doing so, we aimed to encode the increased RS-FC in the music group from 33 to 40 weeks over the control group from 33 to 40 weeks. The conversion of p-values to z-scores was performed by using the quantile function of the normal distribution for which the z-scores follow the standard normal distribution under the null hypothesis, that the groups are equal. After subtracting the z-score of the control group from the music group, the vector of differences was made normally distributed under the null:

$$\frac{{\text{Z}}_{PM}-{\text{Z}}_{\text{P}C}}{\sqrt{2}}∼\text{N}\left(0,1\right).$$

We divided by sqrt(2) to obtain valid z-scores with zero mean and unit standard deviation (based on the Variance Sum/Difference Law we have):

variance(ZPM−ZPC)=variance(ZPM)+variance(ZPC)−2cov(ZPM,ZPC)=2,

assuming that$2\mathrm{cov}\left(Z_{PM},Z_{PC}\right)=0$under statistical independence of group-specific z-score vectors, enabling a z-score threshold corresponding to a specific p-value under the null and obtaining significant results at the specific alpha level (0.05).

## 3 Results

### 3.1 The resting-state functional connectivity maturation from 33 to 40 weeks GA

**Fig. 2.**
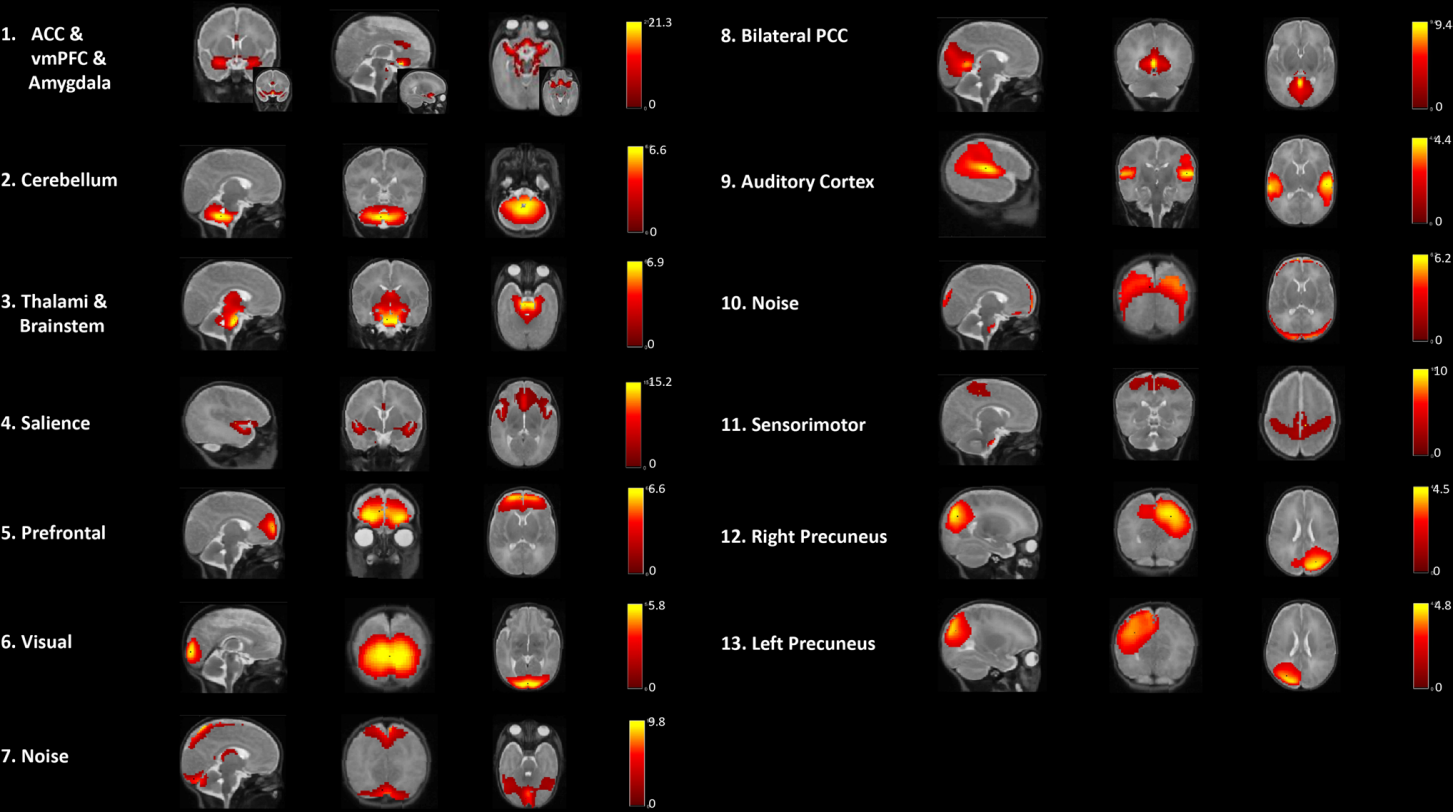
Components obtained from the premature data-driven ICA group analysis at 33- and 40-week GA combined. Each row shows sagittal, coronal, and axial view of the components overlaid onto the 40-week GA template. Images were thresholded at a z-score of 2. The colored bars show the corresponding z-score.

For the developmental exploration from 33 to 40 weeks GA in the sample of preterm infants, an ICA was conducted on the combined resting-state data acquired at 33 weeks GA (mean GA 33.6 weeks (SD = 0.4)) and 40 weeks GA (40.1 weeks (SD = 0.5)).Figure 2shows the results of the combined data-driven ICA. Eleven robust RSNs were found within areas for primary sensory functioning, that is, the sensorimotor, visual, and auditory networks. Associative resting-state activation was found for areas involving the bilateral PCC, and the precuneus, which was provided in separate hemispheric networks. The PCC and precuneus are considered as part of a primitive DMN. One component contained the salience network with insular and ACC activation. Another component comprised the prefrontal cortex (PFC) as part of the central executive network (CEN). A prefrontal–limbic circuitry component involved the regions of ACC, ventral medial prefrontal cortex (vmPFC), and amygdala. Additionally, subcortical areas besides the amygdala involved the cerebellum, thalami, and brainstem.

### 3.2 Connectome of interest at 33 and 40 weeks gestation

The connectome-based analysis of the ICA results treated the ICA spatial components (the 11 RSNs) as nodes and the accordance, or functional connectivity/coupling between these nodes as edges, or connections, in a network representation. Thus, the connections corresponded to the measure of the statistical dependencies between the corresponding time courses of the obtained RSN and can be summarized in a matrix format, that is, the RSN-FC matrix (seeFig. 3).

**Fig. 3.**
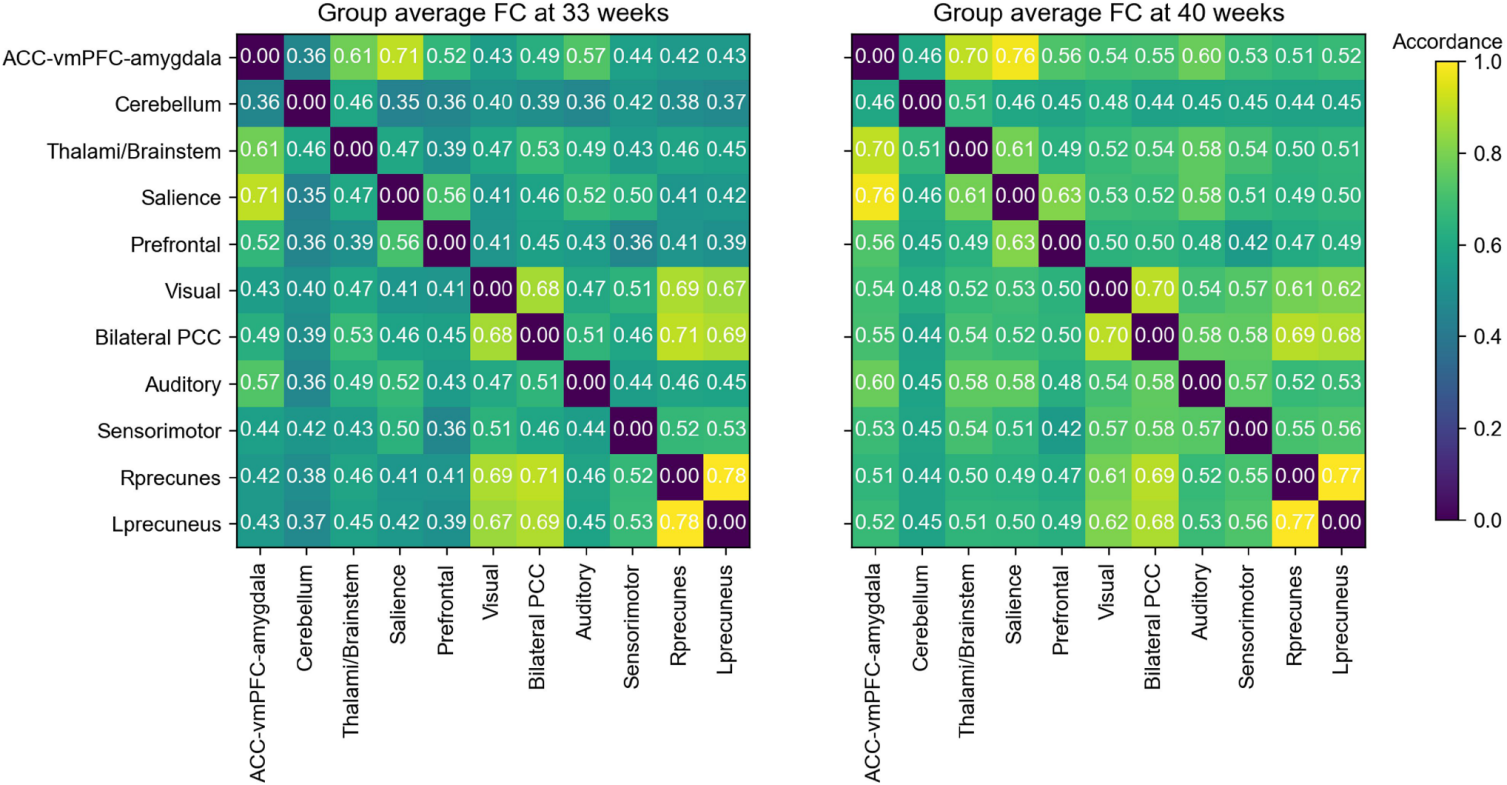
Visual representation of the group-averaged ICA-FC using accordance. The ICA-FC is an 11 x 11matrix representing the connectivity between all possible pairs of networks obtained from the combined group ICA of the preterm infants at 33- and 40-week GA. The left matrix shows the internetwork accordance using the premature infants’ time courses at 33 weeks GA, while the right matrix shows the computed internetwork accordance using the premature infants’ time courses at 40 weeks GA. The values depicted within the matrices represent the mean connectivity across subjects. Higher values in the colored bar depict the level of increased accordance between RSNs. Components are as follows: ACC, vmPFC, amygdala—cerebellum—thalami & brainstem—salience—prefrontal—visual—bilateral PCC—auditory—sensorimotor—right hemispheric precuneus—left hemispheric precuneus. (ACC = anterior cingulate cortex, vmPFC = ventral medial prefrontal cortex, PCC = posterior cingulate cortex, Rprecuneus = right hemisphere precuneus, Lprecuneus = left hemisphere precuneus).

**Fig. 4.**
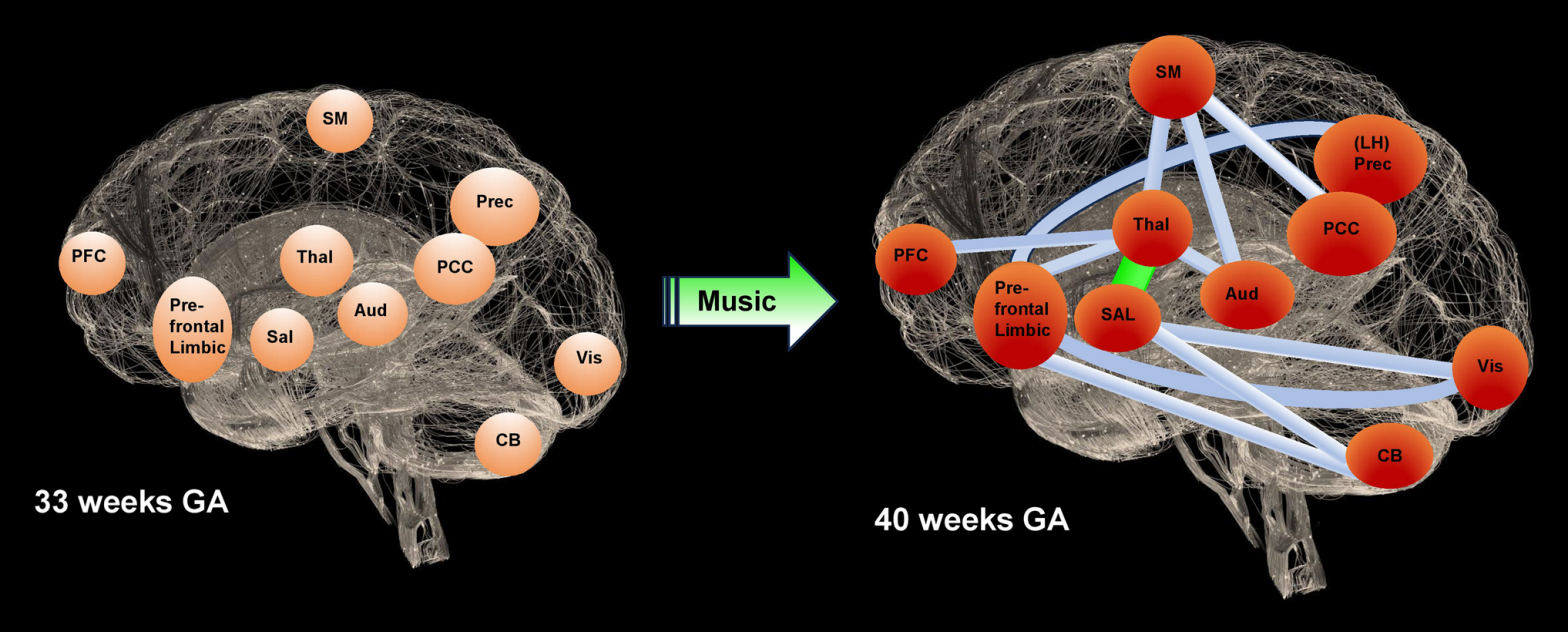
Graphical representation of the music effects on premature RSN maturation from 33 to 40 weeks GA. Based on the ICA results fromTable 1, the 12 connections maturing in RS-FC (FDR < 0.05) are depicted between thalamic/brainstem and salience, prefrontal–limbic, auditory, sensorimotor, and prefrontal cortical networks; between prefrontal–limbic, visual, cerebellar, and left hemispheric precuneus networks; between salience, visual, and cerebellar networks; between the sensorimotor networks, auditory, and bilateral PCC networks. Based on the results fromTable 2, the maturation difference measured in accordance between the music group and the control group is displayed for the connection between the thalamic/brainstem and salience networks. (Thal = thalami & brainstem, Sal = salience, prefrontal–limbic = ACC vmPFC amygdala, SM = sensorimotor, Aud = auditory, Vis = visual, CB = cerebellum, PFC = prefrontal cortex, PCC = posterior cingulate cortex, Prec = precuneus, LH Prec = left hemispheric precuneus).

### 3.3 Circuitry of interest for maturation from 33 to 40 weeks GA

In order to evaluate longitudinal changes in functional connectivity strength, the maturating COI was defined by comparing the ICA-FC matrices for each premature infant at 33 weeks GA to 40 weeks GA as described in Section “*2.7 Connectome-based Statistical Analysis*”.

The maturating COI reflected for the entire sample of preterm infants the increased accordance or RS-FC strength between ICA-derived component connections at 40 weeks GA compared with 33 weeks GA.Table 1shows the t-test results and corresponding p-values prior to and after FDR correction at α = 0.05 for each connection, thus providing the connections with significantly increased accordance at 40 weeks GA. The results revealed significantly increased RS-FC between the thalamic/brainstem network and five networks, namely the auditory network, sensorimotor network, prefrontal network, ACC vmPFC amygdala network, and salience network. The ACC vmPFC amygdala network showed significant increase in RS-FC at 40 weeks GA with the thalamic/brainstem network, visual network, cerebellar network, and left lateralized precuneus network. The salience network significantly increased in RS-FC at 40 weeks GA with the thalamic/brainstem network, visual network, and cerebellar network. And the sensorimotor network exhibited significant increase in RS-FC at 40 weeks GA with the thalamic/brainstem network, auditory network, and bilateral PCC network.

**Table 1.** COI definition based on statistical accordance comparison between premature infants at 40 weeks GA to 33 weeks GA showed 12 connections that survived FDR corrections at α = 0.05.

Region 1	Region 2	p-Value FDR	p-Value original	EWD
Thalami/Brainstem	Salience	0.0104	0.0005	0.1427
Thalami/Brainstem	Sensorimotor	0.0214	0.0020	0.1127
Thalami/Brainstem	Auditory	0.0368	0.0064	0.0934
Thalami/Brainstem	Prefrontal	0.0403	0.0081	0.0994
Thalami/Brainstem	ACC vmPFC Amygdala	0.0331	0.0048	0.0865
ACC vmPFC Amygdala	Visual	0.0329	0.0042	0.1058
ACC vmPFC Amygdala	Cerebellum	0.0368	0.0067	0.1
ACC vmPFC Amygdala	Left Precuneus	0.0403	0.0088	0.0987
Salience	Visual	0.0210	0.0015	0.1212
Salience	Cerebellum	0.0214	0.0023	0.1148
Sensorimotor	Auditory	0.0029	5.40E-05	0.1377
Sensorimotor	Bilateral PCC	0.0104	0.0006	0.1177

EWD = Edge Weight Difference.

There was no longitudinal significant decrease of the RS-FC of any of the connections between RSNs from 33 to 40 weeks GA (Further details on RS-FC group comparisons are described in theAppendix).

### 3.4 Effect of music on maturation from 33 to 40 weeks GA

Based on the two-step methods described in Section “*2.8 Effect of music on maturation from 33 to 40 weeks GA*”, the maturating COI ofTable 1serves as a backbone for the comparison of RS-FC between the music group at 40 weeks GA versus the music group at 33 weeks GA (Fig. 4). The converted p-values from the t-tests into z-scores were thresholded using a z-value of 1.3, which corresponded to a p-value of 0.1.Table 2shows the results regarding the difference in RS-FC maturation from 33 to 40 weeks GA between the preterm music and control groups, with an increased accordance in the music group between the thalamic/brainstem and salience networks (Fig. 4).

**Table 2.** ICA-FC difference for the premature infants exposed to music compared with the control group, measured by p-value to z-score conversion for the development from 33 to 40 weeks GA.

Region 1	Region 2	Z-score difference	p-Value
Thalami/Brainstem	Salience	1.3	0.1

## 4 Discussion

Prematurity affects brain maturation and many studies have convincingly shown alterations of fMRI-based functional connectivity differences in preterm infants at term equivalent age compared with full-term infants but less is known about the intraindividual maturation of functional connectivity in preterm infants and the modulatory effect of care interventions. The present study revealed a longitudinal developmental trajectory of internetwork RS-FC in premature infants from 33 to 40 weeks GA, based on a connectome approach and identified a maturational circuitry of interest. Additional analyses revealed modulatory effects of a music care intervention on RS-FC strengthening, particularly on the thalamic/brainstem and salience network connection.

### 4.1 Preterm infant resting-state functional connectivity maturation

The preterm infants’ 33- and 40-week GA combined ICA provided the RSNs used as nodes for the connectome analyses. The results defined a maturating COI of increased longitudinal RS-FC from 33 to 40 weeks GA for 12 connections between RSNs. Our results showed that the thalamic/brainstem RSN is the most frequently involved in the connections undergoing a significant longitudinal RS-FC increase from 33 to 40 weeks GA in premature infants, being present in 5 out of the 12 connections. It is followed by the ACC-vmPFC-amygdala (prefrontal–limbic) network, involved in 4 out of the 12 connections undergoing significant RS-FC increase. The salience network and sensorimotor networks follow, being involved, each of them, in 3 out of the 12 connections undergoing significant RS-FC increase. The auditory, visual, and cerebellar networks are involved, each of them, in 2 out of the 12 connections undergoing significant RS-FC increase. And the bilateral PCC, left precuneus, and prefrontal networks are involved, also each of them, in 1 out of the 12 connections undergoing significant RS-FC increase.

The observed increased functional connectivity between different RSNs during this period corresponds with typical fetal third trimester development and is in agreement with cross-sectional studies showing increasing brain functional connectivity with increasing gestational age (Eyre et al., 2021;He & Parikh, 2016;Schöpf et al., 2012;Smyser et al., 2010,^2011^,^2016^;Thomason et al., 2013). A maturational trajectory in RS-FC was further confirmed by the absence of reduction in RS-FC from 33 to 40 weeks GA.

#### 4.1.1 Thalamic connectivity

The thalamic/brainstem network shows a longitudinally increased functional connectivity from 33 to 40 weeks GA with 5 other main RS-networks: sensorimotor, auditory, salience, prefrontal networks, and, specifically, prefrontal–limbic networks. Note that the thalamus, as subcortical structure, serves also as a relay to integrate brainstem activity (Mitchell et al., 2014;Moustafa et al., 2017), which can explain our finding of a combined thalamic/brainstem network.

Thalamocortical connections are established during the late second and third trimester of fetal development (Kostovic et al., 2014), and a rapid growth and establishment of functional thalamocortical connections have been shown to occur during this critical period of early brain development (Kostovic and Jovanov-Milosevic, 2006;Krsnik et al., 2017;Pouchelon & Jabaudon, 2014). Indeed, during the third trimester, the thalamic afferents destined to become thalamocortical projections reside in the subplate and start invading the cortical plate becoming a major site of synaptic interactions, mainly in primary sensory and associative cortical regions, that is, auditory, visual, somatosensory, and frontal (Kostovic & Judas, 2007;Kostovic & Jovanov-Milosevic, 2006;Zhang et al., 2010). Our findings, therefore, align with histologic data, proving that this phenomenon is accompanied by a significant increase of functional thalamocortical connectivity within primary sensorimotor, primary auditory, and even associative networks, such as prefrontal–limbic, prefrontal, and salience networks.

Other functional studies have proven an increased thalamic functional connectivity with an increasing GA in preterm infants, namely between the thalamus and primary motor and sensorimotor regions (Thomason et al., 2015;Toulmin et al., 2015). Research byToulmin et al. (2015)showed that the thalamus at term age, that is, in both full-term and preterm infants, was found to contain robust functional connectivity to the sensorimotor network, reporting additionally that the sensorimotor component occupied the largest portion of the thalamus. They explained that the thalamic–sensorimotor connectivity in preterm infants is stronger during earlier stages of preterm development in comparison with that in term-age, suggesting that these projections may strengthen faster over other thalamic connections. An increase in RS-FC between the thalamic–sensorimotor network may also reflect the environmental stimulation due to prematurity.Toulmin et al. (2015)also has shown an increased connectivity with increasing GA between the thalami and the frontoparietal insular cortex, the prefrontal cortex, and the ACC (part of the salience network), which corroborates our findings.

Of interest, the thalamus has already been shown as being among the strongest in functional connectivity, otherwise known as a “hub,” in both premature infants and healthy fetuses aged between 26 and 33 weeks GA (De Asis-Cruz et al., 2020). Additionally, structural connectivity studies have also reported the thalamus as a hub in very preterm infants at TEA, meaning that it plays a central role in the formation of long-range connections, facilitating the integration of disparate brain regions (Sa de Almeida et al., 2021).

Early thalamic functional connectivity in preterm infants aged between 30 and 40 weeks GA, namely with sensorimotor and premotor association cortex, was shown to correlate with later motor and also cognitive development at 20 months of age, proving the clinical importance of the establishment of thalamocortical connectivity during this period of development (Toulmin et al., 2021).

#### 4.1.2 Prefrontal–limbic circuitry

The co-occurrence of amygdala, ACC, and vmPFC as one ICA-derived RSN component present already at 33 weeks GA implies the important function of this prefrontal–limbic circuitry during early brain development. Our results showed an increased RS-FC of this prefrontal–limbic network from 33 to 40 weeks GA to four other networks: the thalamic/brainstem, cerebellar, precuneus, and visual networks. Of notice, limbic regions have been reported as functional hubs as well in full-term newborns (De Asis-Cruz et al., 2015) but less so in preterm infants. Thalamic–anterior cingulate and thalamic–prefrontal connectivity have been shown to increase in preterm infants alongside their increasing GA (Toulmin et al., 2015).

Regarding the observed increased prefrontal–limbic to thalamic connectivity, it can be viewed as an early relay function of the anterior and midline thalamic nuclei between limbic, anterior cingulate, and orbitofrontal regions, and also toward the hippocampus, which is well described in the mature brain (Canini et al., 2020).

Tracing studies have substantiated prominent connections from limbic (amygdala), paralimbic (cingulate), and prefrontal cortices to the cerebellum (Dum & Strick, 2003;Middleton & Strick, 1997,^2001^;Schmahmann & Pandyah, 1997). In adults, resting-state cerebellar connectivity has been shown to be present with the prefrontal cortex and the frontal pole (O´Reilly et al., 2010), as well as with parts of the salience network including the ACC and insula (Habas et al., 2009). Our results indicate maturation of this prefrontal to cerebellar network early in preterm brain development.

Furthermore, our results show an increased RS-FC from 33 to 40 weeks GA between the prefrontal–limbic circuitry and left precuneus network. The precuneus is the core part of the DMN, an important network for social cognition (Mars et al., 2012), inhibitory control (Whitfield-Gabrieli & Ford, 2012), and self-related processes (Fransson & Marrelec, 2008), functions closely related to emotional processing. In the neonatal brain, the left lateralized precuneus has been found to have a high betweenness centrality (Ratnarajah et al., 2013), meaning it integrates as a hub a widespread network in the left hemisphere. Its function involved in conscious perception and episodic memory, integrating experience of sensory information, associates with prefrontal activity to episodic recall related to the self in terms of familiarity processing (Lou et al., 2004;Lundstrom et al, 2005). In recent studies on the structural connectivity in newborn infants, the precuneus appeared as an important structural node but demonstrated decreased structural connectivity both intra- and interhemispheric in preterm infants compared with full-term infants, which indicates its vulnerability in the critical period of development (Sa de Almeida et al., 2021).

Regarding the increased prefrontal–limbic connectivity to the visual network, it could potentially be argued that the period in the NICU is associated with stimuli that otherwise do not occur in-utero. In fact the visual cortex in premature infants has shown to be more strongly interconnected than age-comparable fetuses (De Asis-Cruz et al., 2020) and to present an accelerated electrophysiological and neurobehavioral maturation (Ricci et al., 2008;Schwindt et al., 2018). Of interest, efferent connections from the amygdala are prominently widespread to the primary visual cortex as part of the ventral visual pathway, which indicates a role of the amygdala in vision awareness (Duncan & Barrett, 2007;Pessoa, 2010). The increased functional connectivity in the prefrontal–limbic network and the visual network can reflect, therefore, not only the establishment of this network during early development, but also the effect of early visual input on premature infants’ connectivity and its interconnection with emotional processing.

The increased prefrontal–limbic connectivity between 33 weeks and TEA to all mentioned regions may thus reflect third trimester development, with immature elements of DMN connection formation and integration of environmental stimuli. Furthermore, newborn studies have shown that the amygdala–ACC–vmPFC connectivity predicts fear, sadness, and cognitive development at 6 months of age (Graham et al., 2016;Thomas et al., 2019), denoting the importance of this brain network for future emotional and cognitive functioning.

#### 4.1.3 Salience network

The salience network is recognized for its role in relevance assessment of environmental stimuli and identifying internal appropriate responses (Li et al., 2019;Seeley, 2019;Taylor et al., 2009). Our results showed an increased connectivity between salience nodes and the thalamic/brainstem network, as well as between cerebellar and visual networks from 33 to 40 weeks of gestation. The development of the salience network has been established present over the entire duration of the third trimester of gestation (Afif et al., 2007;De Asis-Cruz et al., 2021;He & Parikh et al., 2016;Ji et al., 2022;Scheinost et al., 2024;Turk et al., 2019).Lordier, Meskaldji, et al. (2019)described the salience network as an important central hub at TEA in both preterm and full-term infants. They showed its connectivity to circuitry involving primary sensory, thalamic, and higher order RSNs, while highlighting that the RS-FC strength appeared compromised for premature infants. Our results revealed a significant developmental trajectory of increased salience–thalamic and salience–visual RS-FC from 33 to 40 weeks GA, relating to the development of centrality of this network, as has been found at TEA byLordier, Meskaldji, et al. (2019). The increase in RS-FC between the visual network and the salience network may reflect stimulation by the environment ex-utero. Of potential interest in terms of long-term developmental effects,Alcauter et al. (2014)found a correlation between visuospatial performance at 1 and 2 years of age and salience–thalamic resting-state connectivity in full-term newborns.

We also reported an increased RS-FC between the salience and cerebellar networks. Indeed, the cerebellar vermis has been found to connect to insular regions (Bernard et al., 2012;Habas et al., 2009;Herzmann et al., 2019). Our results, therefore, provide substantial evidence for a significant functional maturation of this network during early preterm development.

#### 4.1.4 Sensorimotor cortex

Our results revealed an increase in RS-FC strength from 33 to 40 weeks GA between the sensorimotor network, and thalamic/brainstem, auditory, and bilateral PCC networks. As mentioned previously, enhanced sensorimotor–thalamic connectivity is observed during third trimester development (Toulmin et al., 2015). In addition, sensorimotor areas in the newborn brain are the most active, well connected, and dynamic (Huang et al., 2020). In addition, primary sensory local resting-state activity occurs over dynamic connectivity to other regions in newborns (Huang et al., 2020), thus enhanced primary sensory connectivity such as between the sensorimotor–auditory networks is to be expected. The long-range connection to bilateral PCC may reflect a motor component; it is identified that the sensorimotor connectivity to the PCC reflects the movement-readiness phase, by retrieving motor imagery, and suited moving parameters (Treserras et al., 2009).

These results together reflect the important establishment of functional connectivity during preterm brain development, occurring from 33 to 40 weeks GA, between thalamic, prefrontal–limbic, salience, and primary sensory/sensorimotor regions, as well as early components of DMN and cerebellum.

### 4.2 Modulatory effect of music on preterm longitudinal resting-state functional connectivity maturation

A secondary investigation aimed at investigating the modulatory effects of music as environmental enrichment in the NICU on preterm infants´ RS-FC maturation. The modulation of preterm internetwork RS-FC strength after exposure to music can be explained by the finding that spontaneous neural activity (Khazipov & Luhmann, 2006;Penn & Shatz, 1999) and sensory inputs have shown to be required for the establishment of cortical networks maturation during late gestation (Bourgeois et al., 1998;Huttenlocher, 1979). Compared with the control group, the infants exposed to music exhibited a significantly stronger RS-FC between the thalamic/brainstem and salience networks from 33 to 40 weeks GA.

#### 4.2.1 Thalamic–salience integration after music intervention

Our finding that preterm infants exposed to music showed a significantly increased connectivity between the thalamic/brainstem and salience networks from 33 to 40 weeks GA compared with the premature control group is pertinent. Connectome research among preterm infants at term and full-term newborns has shown that the connectivity between the thalamus and salience networks was found to be weaker in preterm infants at TEA compared with full-term newborns (Lordier, Meskaldji, et al., 2019). In their 2019 study, Lordier, Meskaldji, and colleagues demonstrated that a music intervention in the NICU improved the RS-FC within the salience network of preterm infants, achieving levels comparable to those of full-term infants at TEA. They also emphasized the importance of the salience network at term age, noting its role as a central hub in connectivity with other resting-state networks, including connections to the thalamus. In the current study, we now can show that this effect is supported by a longitudinal increase in maturity of this network through the music intervention.

A study byPerani et al. (2010)further supports the effect of music on salience network resting-state activity from the first hours after birth up to 3 days on emotional reactivity, partly through activation in the right insula. Resting-state connectome analyses byLoukas, Lordier, et al. (2022)further revealed that in preterm infants at TEA, music listening affected ACC activity.

Loukas, Lordier, et al. (2022)further related familiarity processing of music through enhanced connectivity between the right amygdala and the right thalamus, the left superior parietal gyrus (precuneus), right temporal pole, and left thalamus, as well as connections from the right orbitofrontal region. Furthermore, diffusion studies using the same music intervention for examining preterm brain structural development found an increased maturation of the orbitofrontal cortex, temporal pole, insula (part of the salience network), and uncinate fasciculus in preterm infants at TEA (Sa de Almeida et al., 2020,^2023^).

An enhanced thalamic-connectivity maturation may provide long-term benefits for the developing brain. Especially, since thalamic connections have been reported to be compromised during early infancy (Nunes et al., 2020), with protracted effects well into adulthood (Bäuml et al., 2015). Furthermore, in terms of potential long-term developmental outcome, visuospatial performance at 1 and 2 years of age has been related to salience–thalamic resting-state connectivity in full-term newborns (Alcauter et al., 2014).

Based on these results, we could infer that enrichment of the extrauterine NICU environment through music stimulation has led to an increased RS-FC between the salience and thalamic networks. This enhancement in preterm brain development is pertinent, as the salience and thalamic networks are undergoing significant maturational changes in RS-FC from 33 to 40 weeks GA. Additionally, these networks serve as central hubs in the connectivity of other developing resting-state networks.

Taken together, we can state that the early functional brain development in preterm infants is characterized mainly by the establishment of thalamic/brainstem, prefrontal–limbic, salience, and primary sensory/sensorimotor connectivity, along with early DMN and cerebellar connectivity. The modulatory effect of music on thalamic–salience network functional maturation indicates that there is room for plasticity during this developmental key period of extrauterine life in the NICU. The impact of music through enhanced thalamic and salience integration may affect the maturation of wider circuitry. Musical stimulation as proposed in the current study can thus be considered an important tool for enhancing brain connectivity during early development.

## 5 Limitations

The template used to compare the 33 weeks with the 40 weeks GA scans is derived from 20 full-term and preterm at TEA infants. Stretching and comparing the 33 weeks GA scans based on a 40-week GA template could compromise the localization of the brain areas given the expected developmental change in shape and volume within the population. A solution to minimize size effects could be to have a mid-way template which arguably is more age appropriate and, therefore, would be more advantageous for the precision. However, our findings concerning each of the two groups at 33 and 40 weeks GA have not been affected due to the fact that the warping is consistent within the group. In addition, the signal intensity is unaffected by the normalization, meaning there is no modulation, and, therefore, the functional connectome is not compromised either.

It could be further argued that a sample larger than n = 43 could yield more power to detect maturational changes, and differences in the music group of n = 24. Reproducibility of the results with larger samples is recommended (Button et al., 2013), considering variations in the ICA results with other studies at term. However, our results are in line with our previous findings of an identical music intervention, showing the impact on RS-FC in another preterm population at TEA, validating the reliability of our results.

In addition, we discuss the results of RS-FC maturation for RSN connections passing statistical significance when correcting for multiple comparisons. Slight differences observable in the group-averaged ICA-FC at 40 weeks GA compared with 30 weeks GA may be still of interest to explore. Perhaps not readily feasible for the discussed population, however, rs-fMRI scan acquisition at more than two measurement time points could elucidate in more detail the maturational changes in the preterm infant group in its entirety, and show particularly the differences in the music group.

Lastly, while network strengthening is expected to increase over the period of the third trimester, it is well recognized that premature infants at term equivalent age have not caught up to full-term RS-FC strength. Environmental enrichment studies aim to target the development of the preterm brain and have shown to impact the brain at term (Pino et al., 2023). It is important to assess both the impact on development through pre- and postintervention brain data acquisition and alongside the maturation that occurs within preterm infants, keep including full-term comparison in order to reveal the extent of enhanced network strengthening.

### 5.1 Future recommendations

MRI data acquisition is a safe tool for measuring brain development and music can be considered easily applied and inexpensive to enrich the environment and adjust to the needs of premature newborns. This makes inclusion of larger samples for future studies accessible, which is recommended to assess developmental trajectories and to investigate the music effects on premature brain maturation. Since music aims to aid in early neurodevelopment and plasticity to lay the foundation for future functioning, following up after discharge through neurodevelopmental assessment can further reveal the effects of NICU music intervention.

## 6 Conclusion

We explored premature newborn internetwork resting-state brain connectivity maturation from 33 to 40 weeks GA, based on the accordance between data-driven derived ICA networks. The functional connectivity strength between RSNs increases for brainstem/thalamic, prefrontal–limbic, salience, and sensorimotor areas with primary sensory, associative, and subcortical regions. Under a music intervention, there is enhanced strengthening of RS-FC, which provides evidence that an enrichment of the environment by music can enhance brain maturation and thus plasticity in the preterm brain.

## Data Availability

The authors confirm that the data supporting the findings of this study are available within the article and inAppendix Table 1. Further details on data availability and code for data analysis in Python can be shared upon request to the corresponding author at the following e-mail address:petra.huppi@hcuge.ch.

## Appendix. Equality of Experimental and Control Groups

As shown in the table, group comparisons reveal no significant differences regarding characteristics associated with premature birth and usable MRI data within the experimental music and control groups.

Additionally, the accordance, or RSN-FC, is tested between the music and control groups at 33 weeks GA, to determine whether either group has more internetwork strength between the components. The accordance does not significantly differ between the music and control group at 33 weeks GA. For maturational assessment prior to the intervention at 33 weeks GA, no significant results were found when testing the accordance of the music and control groups combined at 33 weeks GA, over the accordance of the music and control groups combined at 40 weeks GA.

**Appendix Table A1.** Table on descriptives of group characteristics and differences between premature experimental music and control group.

Means or relative proportions for preterm music and control (n = 43)	Preterm music n = 24 (55.8%)	Preterm control n = 19 (44.2%)	Min–max 1 ^st^ and 3 ^rd^ Quartile	Shapiro–Wilk significance <0.05	Group comparisons
Gestational age at birth (weeks), mean (SD)	29.2 (±1.9)	28.9 (±2.5)	24.1–32.6 27.7–31	W = 0.93	(PM)Mdn = 28.5 (PC)Mdn = 29.1 U = 215, *p* = 0.75
Birth Weight (grams), mean (SD)	1273 (±454)	1134 (±340)	600–2135 920–1550		*t* (40.9) = -1.15, *p* = 0.25
Birth Height (centimeter), mean (SD)	38.7 (±4.1)	37.6 (±3.7)	31.5–46 36–40.75		*t* (40.9) = -1.03, *p * = 0.3
Birth head circumference (centimeter), mean (SD)	26.4 (±2.6)	26.1 (±2.5)	21–30.5 24–28.25		*t* (39.3) = -0.41, *p * = 0.67
Female, n (%)	10/24 (41.6%)	12/19 (63.1%)			χ ^2^ (1, *N =* 43) = 1.9, *p * = 0.27
Twin, n (%)	10/24 (23.3%)	7/19 (16.3%)			χ ^2^ (1, *N =* 43) = 0, *p * = 0.9
Apgar scores 5 minutes after birth	7.9 (±2.5)	7.8 (±2.0)	1–10 7–9	W = 0.82	(PM)Mdn = 8.5 (PC)Mdn = 9 U = 234, *p * = 0.89
Arterial Ph	7.26 (±0.07)	7.25 (±0.07)	7–7.4 7.2–7.3		*t* (39.3) = -0.54, *p * = 0.59
Bronchopulmonary dysplasia, n (%)	7/24 (16.3%)	6/19 (13.9%)			χ ^2^ (1, *N =* 43) = 0, *p* = 1
Early and late onset sepsis, n (%)	4/24 (9.3%)	6/19 (13.9%)			χ ^2^ (1, *N =* 43) = 0.61, *p * = 0.43
Socioeconomic score (range 2–12), mean (SD)	4 (±2)	4 (±2)	2–12	W = 0.75	(PM)Mdn = 3 (PC)Mdn = 3 U = 240, *p* = 0.52
Discharge (weeks), mean (SD)	39.1 (±2.7)	38.4 (±2.3)	35.2–46.4 36.5–40.3	W = 0.92	(PM)Mdn = 38.4 (PC)Mdn = 37.7 U = 191.5, *p* = 0.37
Number of music/no-music intervention, mean (SD)	55.8 (±24.9)	58.1 (±28.4)	14–113 39–70		*t* (32.2) = 0.25, *p* = 0.8
Days of music/no-music intervention, mean (SD)	28.4 (±10)	25.9 (±10.6)	9–47 19–34		*t* (33.3) = -0.74, *p* = 0.46
Gestational age at scan prior intervention (weeks), mean (SD)	33.7 (±0.5)	33.7 (±0.4)	32.8–34.5 33.4–34		*t* (40.9) = 0.17, *p* = 0.86
Gestational age at scan postintervention (weeks), mean (SD)	40.1 (±0.6)	40.1 (±0.5)	38.7–41 40–40.5	W=0.95	(PM)Mdn = 40.1 (PC)Mdn = 40.1 U = 214.5, *p* = 0.74
Number of usable scans prior intervention, mean (SD)	472.8 (±87.9)	471.3 (±85.7)	303–590 398.5–551		t(39) = -0.06, p = 0.95
Number of usable scans postintervention, mean (SD)	520.3 (±77.3)	511.6 (±66.2)	317–590 473–571		t(40.7) = -0.4, p = 0.69

Shapiro–Wilk test shows that five variables are not normally distributed, therefore, consequently the music and control groups’ medians are compared with the Mann–Whitney—U test, and for the normally distributed variables, the music and control groups’ means are compared through parametric t-test.

SD = standard deviation, (PM) = music group, (PC) = control group, Mdn = median.
